# Digital fabrication of customized nasal conformers for unilateral cleft lip repair: a pilot randomized controlled trial

**DOI:** 10.1186/s12903-026-08274-x

**Published:** 2026-04-15

**Authors:** Mahmoud Akram Khodir, Saeeda Mahmoud Osman, Hala Ragaa Ragab, Mamdouh Ahmed AboulHassan, Mona Samy Oraby

**Affiliations:** 1 Oral and Maxillofacial Surgery department, Faculty of Dentistry, Alexandria University, Champlion st, El-Azarita, Alexandria, Egypt; 2 Plastic Surgery department, Faculty of Medicine, Cairo University, Al-Saray St, Al-Manial, Cairo, Egypt

**Keywords:** Digital design, Cleft lip repair, Digital fabrication, Fisher technique, Nasal conformer

## Abstract

**Background:**

Proper nasal repair and relapse prevention remain key challenges in complete cleft lip patients. This study aimed to assess the effectiveness of customized nasal conformers, designed through an easy and reproducible method, in improving nasal symmetry.

**Materials and methods:**

Fourteen patients were enrolled in a pilot randomized controlled clinical trial divided equally into study and control groups. All underwent Fisher anatomic subunit repair. In the study group, a customized nasal conformer was digitally fabricated, applied on the day of surgery, and sutured in place for 3 months. No conformer was used in the control group. Digital anthropometric measurements were performed at 6 months postoperatively, including alar width, alar base width, columella length, nasal tip projection, columellar angle, and nostril height/width ratio. Statistical analyses were performed for both within-group and between-group comparisons and parental satisfaction was assessed using a 10-point visual analogue scale (*p* < 0.05).

**Results:**

The study group showed no significant change in most parameters. The control group showed significant changes in all parameters except nasal tip projection. Symmetry correlation was significantly better in the study group. Between-group analysis showed significantly less deviation in the study group for all parameters except nasal tip projection and columella angle. Comparison of visual analogue scale between 2 groups showed statistically significant difference.

**Conclusions:**

Customized nasal conformers, when employed in unilateral cleft lip repair, showed promising potential in reducing relapse, improving nasal symmetry, refining nasolabial esthetics, and enhancing parental satisfaction.

**Trial registration:**

The trial was retrospectively registered (NCT06637488) on 1st of October 2024 at ClinicalTrials.gov.

## Background

Cleft lip and/or palate (CLP) represents the most widespread birth defect globally [1]. CLP affects not only patient’s physical health but also imposes financial burdens on their families. Prenatal diagnosis has contributed to a reduction in the incidence of CLP among newborns [2, 3]. Despite this progress, increasing living standards have elevated aesthetic requirements. Secondary facial deformities associated with CLP, including nasal and labial malformations, often persist after primary repair. Primary surgical interventions aim to correct these clefts and deformities, yet disparities in the growth of bilateral tissues can occur as the child grows [4, 5]. Patients may experience residual scars and facial asymmetry, which can cause social distress and reduce satisfaction with dentofacial appearance [6]. Thus, the primary objective of treatment is to enable patients to lead healthy and fulfilling lives without their deformity interfering with daily social interactions [7].

Although surgical treatment for unilateral CLP aims to repair lip and nasal deformities, restore symmetry, and support long-term midfacial growth, outcomes may vary due to individual anatomical differences. Each patient’s unique anatomical deformities necessitate individualized surgical approaches where conventional surgical techniques usually fail to address the deformities of nasal cartilage and soft tissue. This may necessitate secondary corrective procedures to address persistent asymmetries [8]. Several presurgical techniques have been proposed to facilitate surgical procedures and improve final shape of nose and lip. These include lip taping and silicone nasal conformers that can be applied after birth. Lip taping aims to approximate the alveolar segments and reduce tension during primary surgery [9]. Silicone conformers help in lifting and reshaping deformed nasal cartilage [10]. Another commonly used method is the nasoalveolar molding (NAM) that is utilized to enhance symmetry of the nose and lip, decrease the severity of the cleft deformity and facilitate primary surgical intervention [11, 12].

Postoperative nasal outcomes may be further enhanced through the use of nasal conformers. These appliances help preserve nostril and septal symmetry, maintain airway patency and ventilation by supporting the alar cartilage and nostril opening, and reduce the risk of hematoma or fibrotic scar formation. Additionally, they assist in the stable positioning of endonasal flaps [13]. Several types of conformers are used worldwide and have been described in literature. Some surgeons use ready-made conformers like the use of two flexible silicone stents connected with a columellar Bridge, others use part of feeding tube or cannula as conformers [14–16]. Recently, custom made conformers have been introduced using various fabrication techniques and materials. The debate remains on how long should the conformer be placed, what are the long term follow up results and what is the best type and methodology of fabrication. Challenges may also arise in achieving parental compliance with the prescribed use of nasal conformers. Moreover, the literature provides limited evidence regarding the potential disadvantages of currently available conformer designs [17]. Some authors have suggested analyzing normal nasal dimensions followed by computer-aided design and 3D printing of the conformer, then the printed part is used to design a mold for casting [18].

Subjective evaluation of surgical outcomes is essential in cleft care, as parental perception often influences overall satisfaction with treatment. The Visual Analogue Scale (VAS) has been widely used as a simple, valid, and reproducible tool for quantifying subjective perceptions of symmetry and aesthetics in craniofacial surgery [19, 20]. Its application in assessing nasal appearance provides a standardized approach for comparing outcomes across different interventions.”

The objective of this study was to assess the effectiveness of customized nasal conformer designed for each patient using an easy and reproducible method to improve symmetry between both sides and enhance esthetics.

The study’s null hypothesis stated that customized nasal conformers would have no significant effect on improving symmetry between the cleft and non-cleft sides.

## Materials and methods

### Study design and ethical approval

This pilot randomized controlled clinical trial was carried out between January and September 2024. Approval was obtained from the Research Ethics Committee, Faculty of Dentistry, Alexandria University, Egypt (IRB No. 001056 – IORG 0008839). The study was performed in accordance with the Declaration of Helsinki [21]. The trial was reported in accordance with the CONSORT 2010 guidelines for parallel-group randomized controlled clinical trials [22], and retrospective registration was completed at ClinicalTrials.gov on 1st of October 2024 (ID: NCT06637488).

### Study setting and participants

The study was carried out in the Oral and Maxillofacial Surgery department, Faculty of Dentistry, Alexandria University. Participants were infants of either gender, with approximate mean age of 4 months, with complete unilateral cleft lip and meeting the “rule of 10s” criteria for primary repair [23]. Exclusion criteria included patients with syndromes and those with a prior cleft lip surgery.

### Sample size calculation, randomization and allocation

Sample size estimation was performed to support feasibility assessment and to provide preliminary estimates of effect size and variability for future trials, rather than for definitive hypothesis testing. Calculation was based on 95% confidence level and 80% study power. Based on previously reported columellar deviation (primary outcome) mean values of 30.74 ± 7.24 mm in patients managed with nasoalveolar molding and 49.31 ± 10 mm in controls [24], the highest SD (10) was used to ensure adequate power. Using Rosner’s method [25] calculated by G*Power 3.1.9.7 [26]. A minimum of six patients per group was required (effect size 1.857). This number was increased to seven per group to account for potential loss to follow-up, yielding a total of 14 participants.

Eligible patients were randomly assigned in a 1:1 ratio to the study or control group using a computer-generated random sequence. Block randomization with block size of two was employed to ensure balanced group sizes [27]. Allocation was concealed using sequentially numbered, sealed, opaque envelopes prepared and handled by an independent researcher not involved in participant enrollment or outcome assessment.

## Surgical procedure

### Presurgical preparation

#### Digital workflow for conformer fabrication

**Fig. 1:**
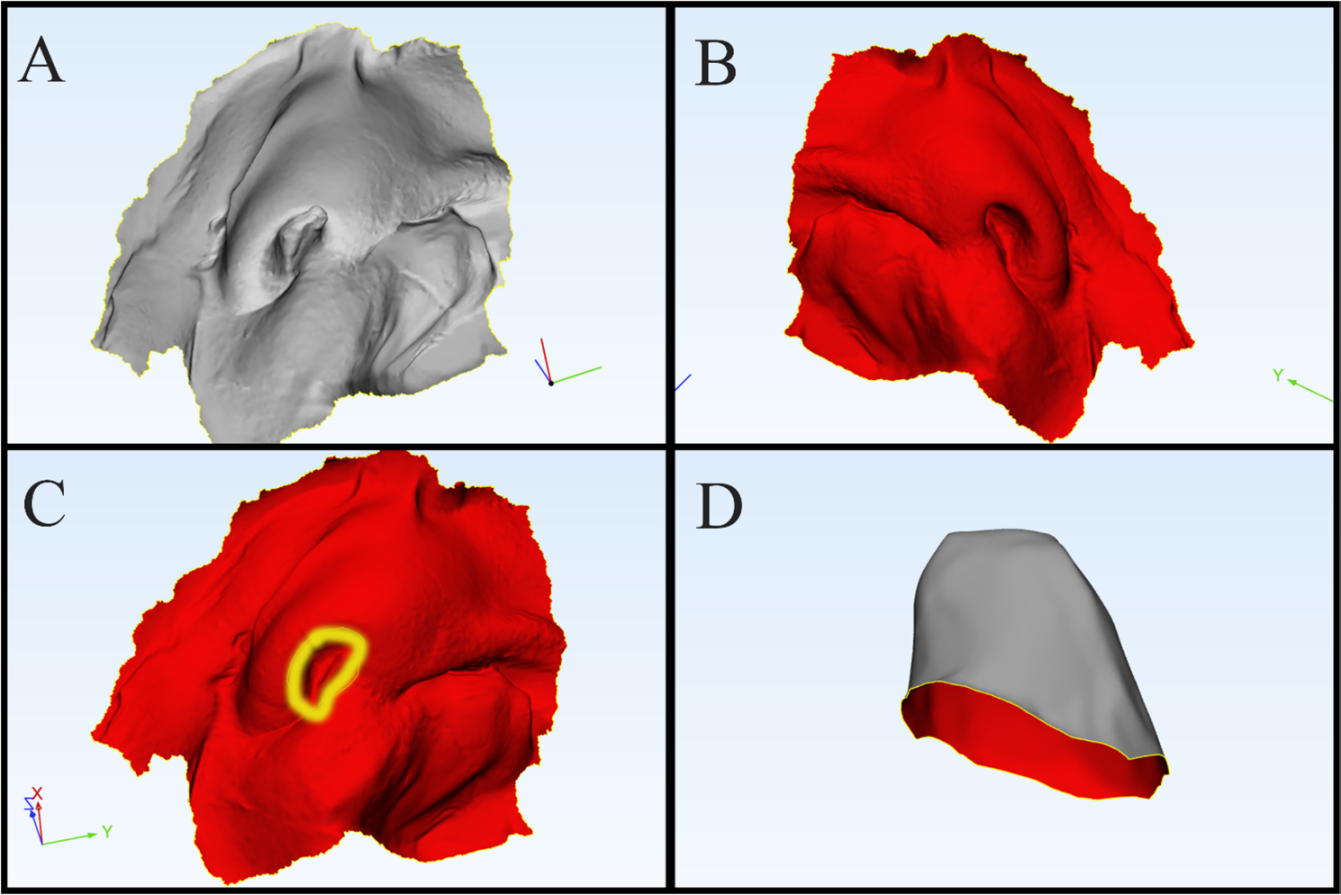
**A** Negative digital model showing impression extension into normal nasal cavity, **B** Positive digital model of nose and lip, **C** Mirror image of the digital model with marked normal nostril contour, **D** Trimmed nostril and nasal cavity with creating 1mm offset

A silicone impression of the nose and lip was obtained using putty addition silicone (Silibest; BMS), with adequate penetration into the normal nasal cavity. The impression was scanned to generate a negative digital model, which was saved as a standard tessellation language (STL) file (Fig. 1A). 

**Fig. 2:**
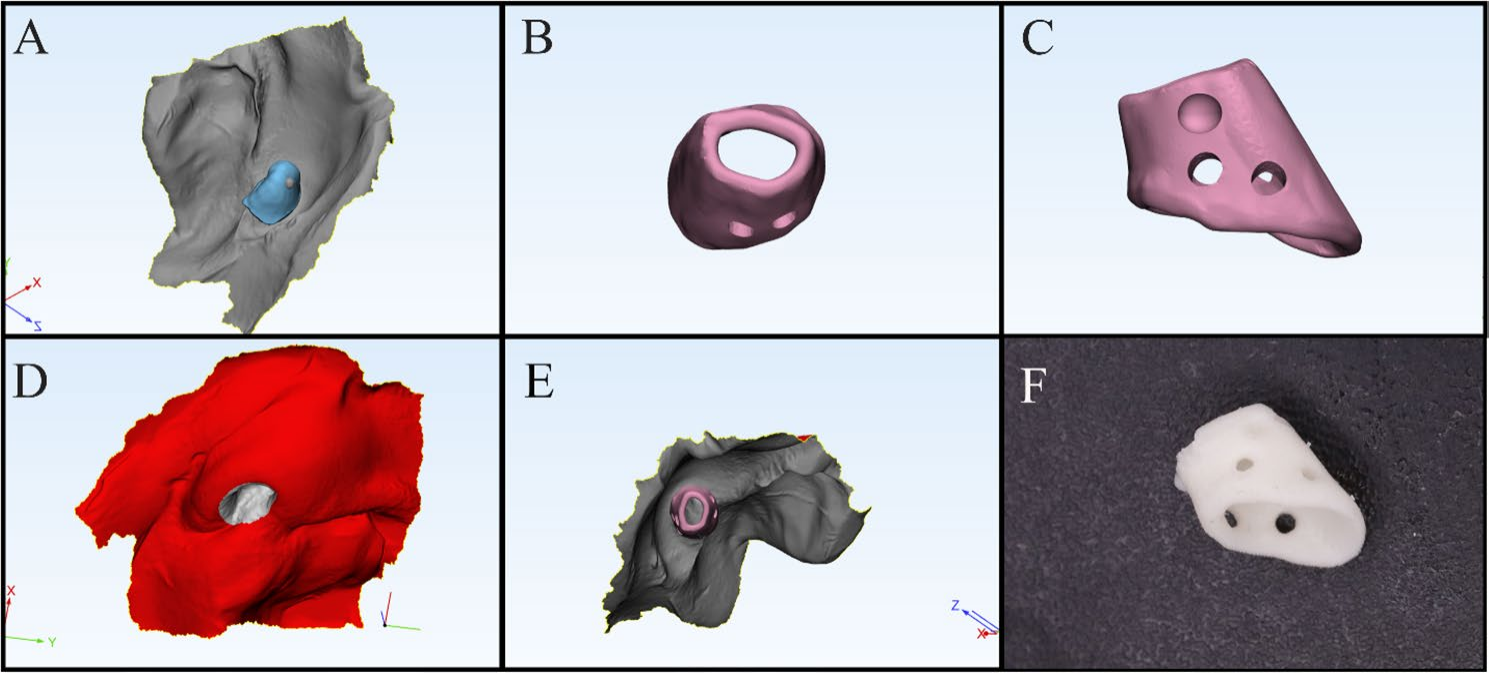
**A **Checking its place on the mirrored model, **B **Conformer hollowed and 2 holes created on each side,** C** Key indentation mark for surgeons, **D** Checking the virtual position of the conformer before printing, **E **Checking virtual position inside the nasal cavity, **F **Printed nasal conformer

The STL file was imported into 3-matic software (Mimics Innovation Suite; Materialise). Using the positive rendering of the model (Fig. 1B), a mirror image of the unaffected side was generated (Fig. 1C). Surfaces surrounding the mirrored nostril were trimmed to replicate the internal nasal cavity and a 1-mm offset was applied to account for anticipated postoperative shrinkage and fibrosis (Fig. 1C, 1D). Additional modifications included creating two holes in the medial and lateral surfaces for suture placement, trimming the superior border to produce a hollow tube and adding a key indentation mark to guide orientation for surgeons intraoperatively. The final design was verified in its virtual position before fabrication (Fig. 2A, 2B, 2C, 2D, 2E).Conformers were printed from polymethyl methacrylate (PMMA) using a 3D printer and disinfected intraoperatively with Lysoformin for 30 minutes (Fig. 2F) [28]. 

All patients received standard preoperative laboratory tests, including hemoglobin level and bleeding profile assessment, and were administered a single intravenous dose of prophylactic antibiotics of Cefotaxime (Cefotax) according to body weight to minimize the risk of postoperative infection.

Written informed parental consent was obtained for the publication of this identifiable image.

### Operative procedure

#### Surgical procedure and conformer placement

**Fig. 3:**
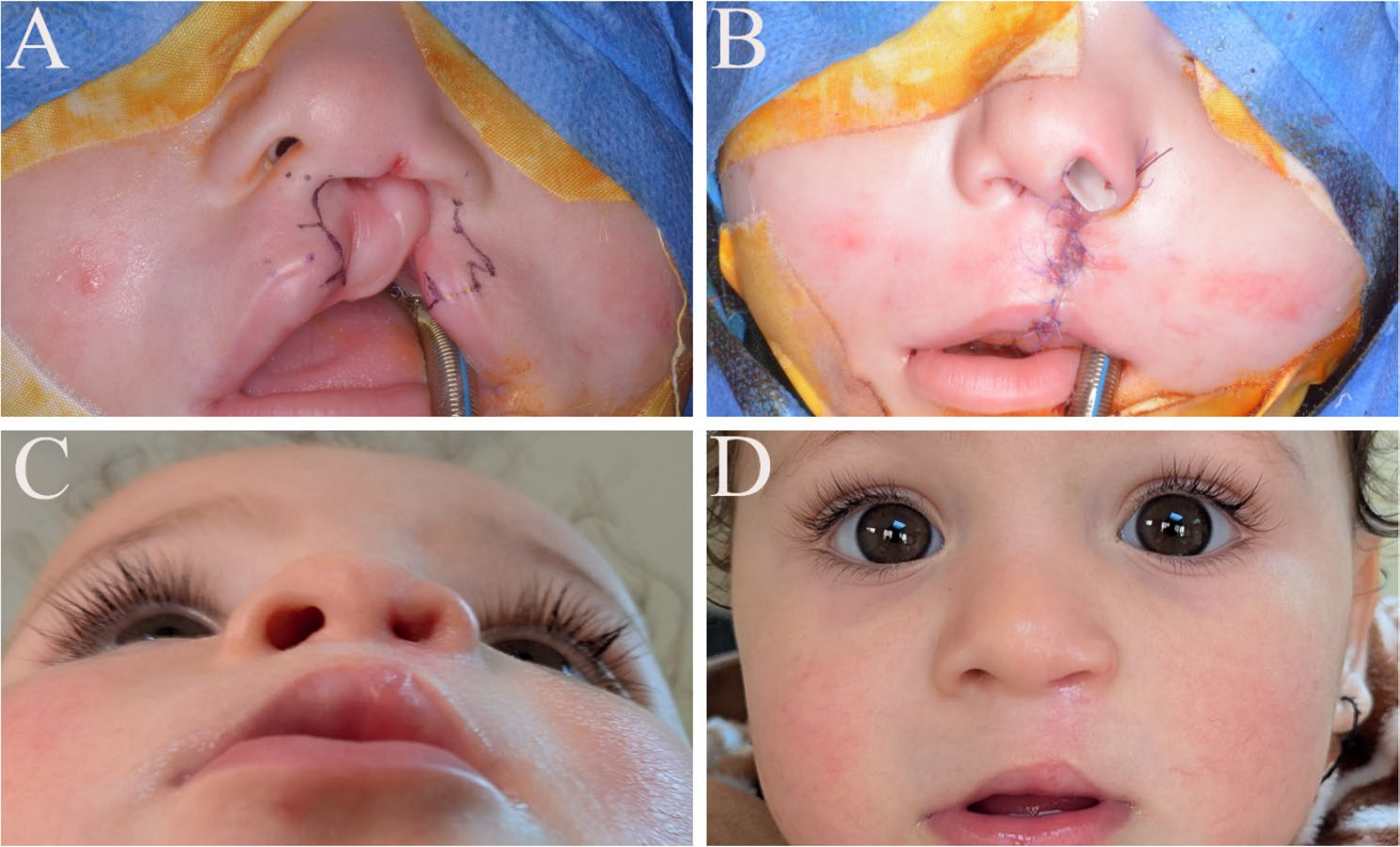
Study group, Case 1 (**A**) Preoperative photo showing markings for Fisher technique (**B**) Immediate postoperative showing conformer in place (**C**,**D**) Six months follow-up. Written informed parental consent was obtained for the publication of this identifiable image

**Fig. 4:**
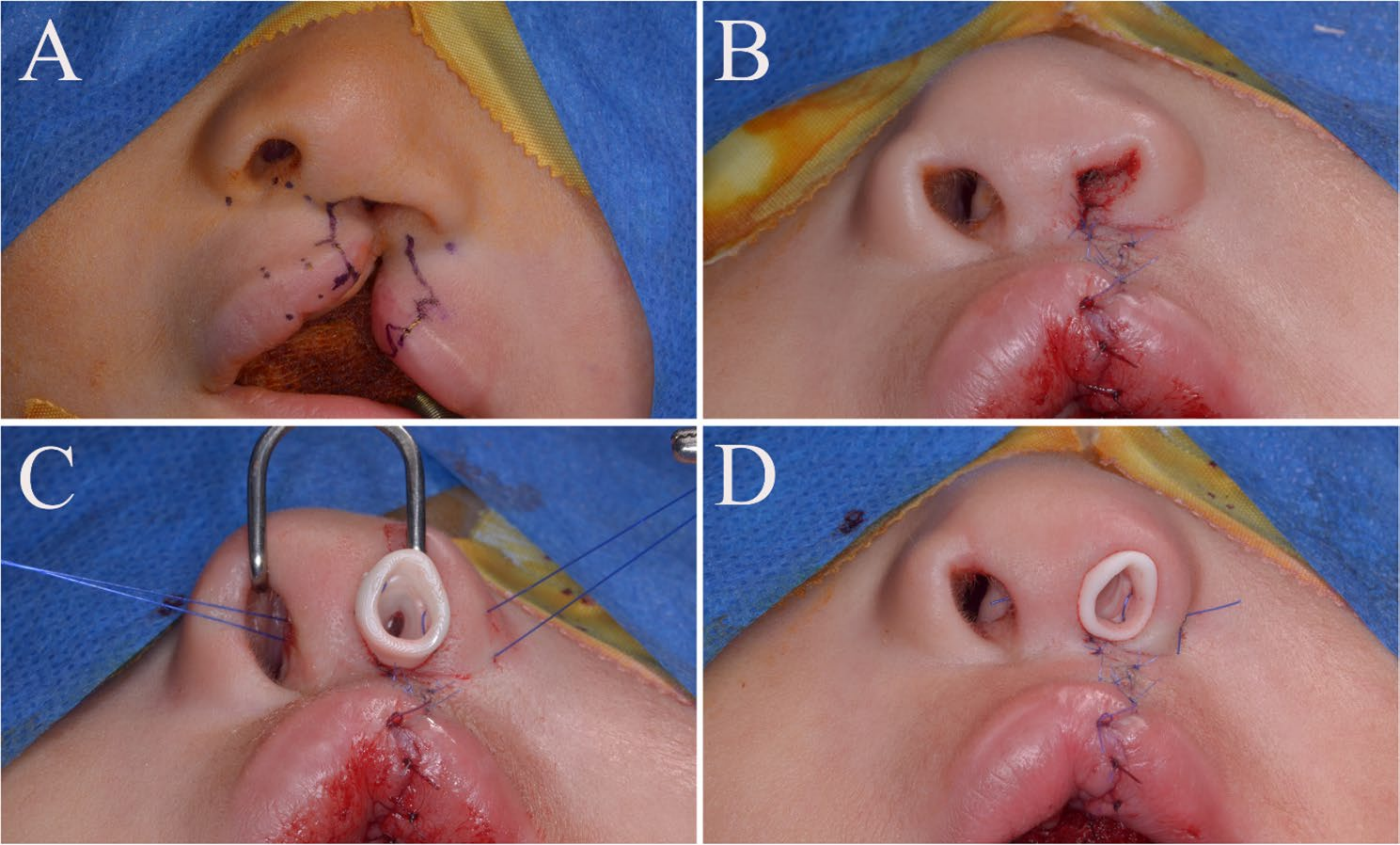
Study group, Case 2 **(A) **Preoperative photo showing markings for Fisher technique **(B)** Immediate postoperative before application of conformer **(C) **Placement process of conformer **(D)** Nasal conformer sutured in place

**Fig. 5:**
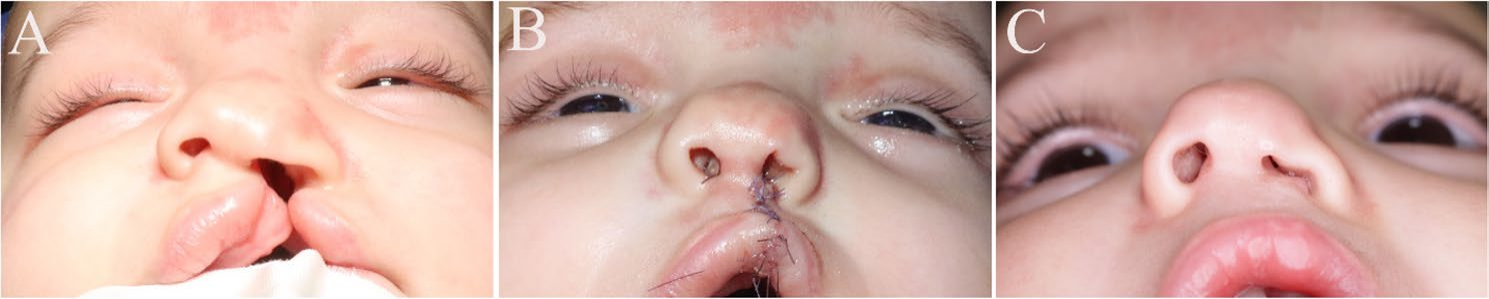
Control group** (A) **Preoperative photo of complete unilateral patient **(B)** Immediate postoperative photo without application of a conformer **(C) **Six months follow-up photo. Written informed parental consent was obtained for the publication of this identifiable image

Both groups underwent cleft lip and nasal repair using the Fisher technique as previously described [29]. Markings were performed with surgical markers to preserve anatomical landmarks, and calipers were used for precise measurements. Incisions and dissections followed these markings, and repair was carried out in three layers: mucosa, muscle, and skin. Closure was performed in layers using interrupted 5-0 Vicryl sutures for the mucosa and muscle, and interrupted 6-0 Prolene sutures for the skin. In the study group, the digitally fabricated nasal conformer was positioned intraoperatively and secured with two 4-0 Prolene sutures: one inter-septal medially and the other through the lateral ala (Fig. 3, 4). The conformer was left in place for three months before removal. The control group underwent lip repair without conformer placement (Fig. 5). 

### Postoperative care

Infants were fitted with arm restraints to prevent disruption of the surgical repair. For the first five days, breastfeeding was avoided and feeding was provided exclusively via syringe. Postoperative medications included cefotaxime (25 mg/kg, intramuscularly, every 12 h for 5 days), topical gentamicin cream (2 cc, three times daily), and paracetamol drops (2.5 cc, up to four doses per day as needed).

### Follow-up protocol

Patients were monitored for six months with three scheduled follow-ups: an early visit at 7 days for suture removal, an intermediate visit at 3 months for conformer removal in the study group, and a final visit at 6 months for impressions to evaluate nasal symmetry and study outcomes.

### Outcomes variables

#### Anthropometric measurements [30] (Fig. 6)

**Fig. 6:**
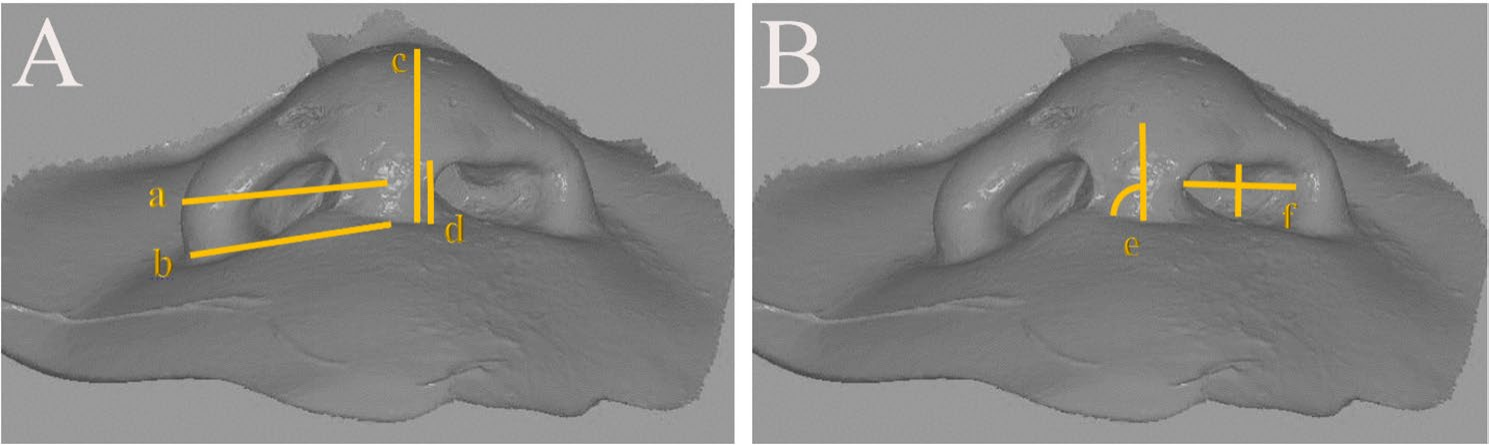
Anthropometric measurements on digital scans (**A**) a- Alar width at maximum convexity b- Total alar base width c- Nasal tip projection d- Columella length (**B**) e- Columellar Angle f- Nostril height/width ratio

Six months postoperatively, nasal symmetry was assessed on digital scans of the final impressions using 3-Matic software (Materialise, Belgium), which included:

- Alar width at maximum convexity.
- Total alar base width.
- Nasal tip projection.
- Columella length.
- Columellar Angle.
- Nostril height/width ratio.

#### Visual analogue scale

**Fig. 7:**
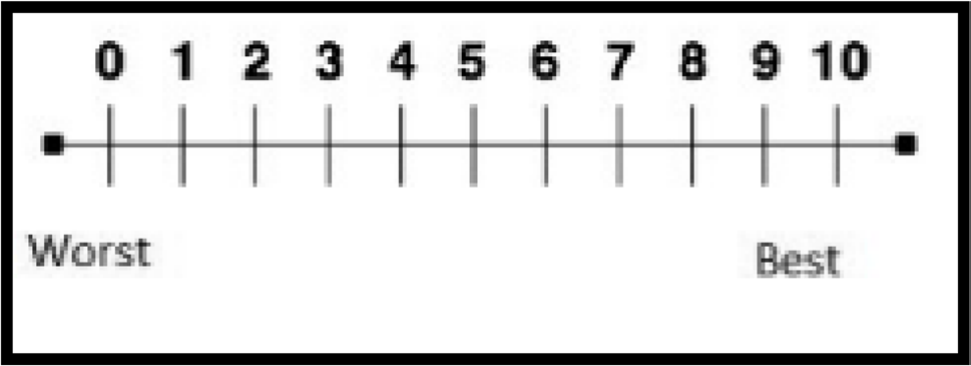
Visual analogue scale

Parental assessment of nasal symmetry was recorded using a 10-point Visual Analogue Scale (VAS), where 0 indicated worst symmetry and 10 indicated perfect symmetry (Fig. 7). Each parent was asked to rate the nasal appearance of their child 6 months postoperatively. The VAS has been previously validated for measuring subjective aesthetic outcomes in cleft and craniofacial research [31]. 

## Statistical analysis

The Shapiro–Wilk test was applied to assess the normality of linear and angular measurements. While both measurement types followed a normal distribution, the mean differences between cleft and non-cleft sides showed non-normal distribution. Data were expressed using mean, median, standard deviation (SD), and interquartile range (IQR). Paired t-tests were applied for within-group comparisons, whereas deviations in linear and angular measurements between the study and control groups were analyzed with the Mann–Whitney U test. Pearson correlation analysis was employed to evaluate associations between cleft and non-cleft side measurements. Intra-examiner reliability was determined using the intraclass correlation coefficient (ICC). VAS scores were analyzed using the Mann–Whitney U test. All tests were two-tailed, with a significance threshold of *p* < 0.05. Statistical analyses were carried out using IBM SPSS Statistics for Windows, version 23 (IBM Corp., Armonk, NY, USA).

## Results

**Fig. 8:**
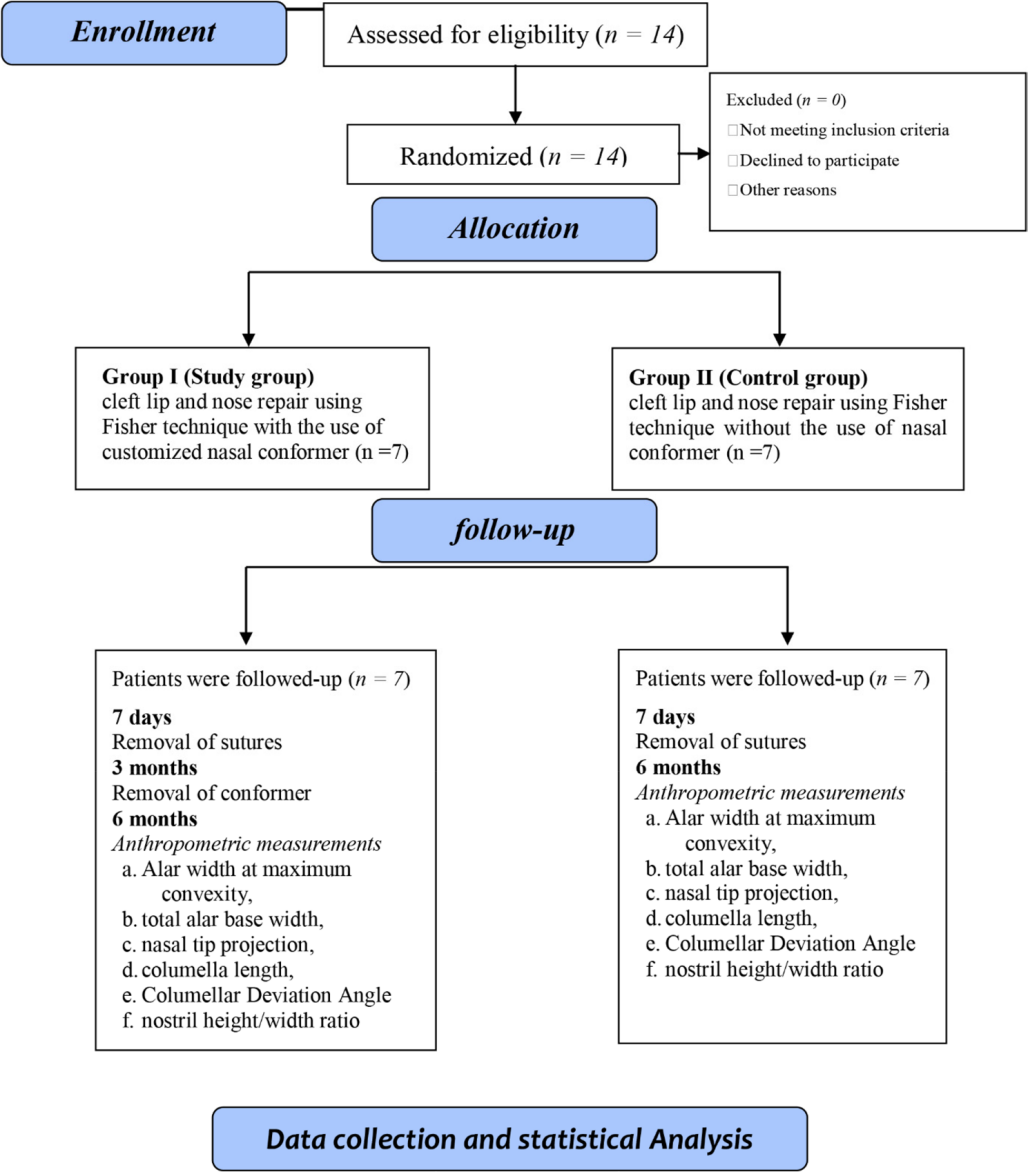
Flow diagram of the randomized trial in accordance with CONSORT guidelines

A total of fourteen patients were enrolled and randomly assigned into two equal groups of seven, with balanced gender distribution. The mean age of the study group was 15.58 ± 2.82 weeks, compared to 17.58 ± 3.16 weeks in the control group, with no significant differences observed in demographic variables. Baseline cleft severity was retrospectively assessed using the Smile Index severity scale (cleft width (CW)/intercommissural distance (ICD)), as described by Yao et al. [32], to confirm comparability between both groups and yields non-significant difference (Table 1). The CONSORT flow diagram of the randomized controlled trial is shown in (Fig. 8).

**Table 1:** Demographic data of the participants

	Study group	Control group	*p* value
Age in weeks (Mean ± SD)	15.58 ± 2.82	17.58 ± 3.16	0.235
Gender: *n* (%)			
Males	3 (42.9%)	4 (57.1%)	1.00
Females	4 (57.1%)	3 (42.9%)	
Cleft side: *n* (%)			
Right	2 (28.6%)	2 (28.6%)	1.00
Left	5 (71.4%)	5 (71.4%)	
CW/ICD Ratio (Mean ± SD)	0.50 ± 0.05	0.50 ± 0.04	1.00

In the study group, anthropometric comparisons between the cleft and non-cleft sides revealed no significant differences across measurements, except for the height-to-width ratio, which demonstrated a significant difference. The control group showed significant difference for all measurements except for nasal tip projection that showed no significant difference (Table 2). Pearson correlation analysis in the study group demonstrated a statistically significant association, indicating greater symmetry between the cleft and non-cleft sides (Table 3).

**Table 2:** Comparison of linear and angular measurements between study and control groups

Variables (in mm)	Study Group			Control Group		
	Non cleft side	Cleft side	*p* value^1^	Non cleft side	Cleft side	*p* value^1^
	Mean ± SD			Mean ± SD		
Alar width at max convexity	14.69 ± 1.95	14.97 ± 1.48	0.533	14.79 ± 1.95	17.21 ± 1.38	< 0.001*
Total alar base width	16.53 ± 2.06	16.77 ± 1.64	0.488	16.63 ± 1.49	19.89 ± 1.69	0.001*
Columella length	4.77 ± 1.21	4.63 ± 1.16	0.648	6.94 ± 1.37	4.84 ± 0.83	0.003*
Nasal tip projection	12.46 ± 1.61	12.56 ± 1.71	0.609	16.33 ± 2.01	15.76 ± 1.89	0.060
Nostril height/width ratio	0.61 ± 0.16	0.55 ± 0.12	0.037*	0.94 ± 0.25	0.43 ± 0.11	0.002*

Bold values indicate statistically significant differences

*Significance was set at a *p*-value < 0.05: Paired t test

**Table 3:** Comparative correlation of linear and angular measurements in study and control groups

Variables	Study Group		Control Group	
	*r*	*p* value	*r*	*p* value
Alar width at max convexity	0.81	0.027*	0.96	0.001*
Total alar base width	0.91	0.004*	0.58	0.175
Columella length	0.78	0.039*	0.53	0.221
Nasal tip projection	0.96	0.001*	0.95	0.001*
Nostril H/W ratio	0.940	0.002*	0.060	0.898

Bold values indicate statistically significant differences

* Significance was set at a *p*-value < 0.05, *r*: Pearson correlation coefficient

Statistical comparison of deviations between study and control groups demonstrated significant differences in all parameters apart from nasal tip projection and columella angle (Table 4).

**Table 4:** Measurement deviation comparison between study and control groups

Variables (in mm)	Study Group		Control Group		*p* value^1^
	Mean ± SD	Median (IQR)	Mean ± SD	Median (IQR)	
Alar width at max convexity	0.29 ± 1.14	0.20 (1.70)	2.43 ± 0.74	2.50 (1.40)	0.005*
Total alar base width	0.24 ± 0.87	0.40 (1.20)	3.26 ± 1.47	2.90 (1.40)	0.002*
Columella length	-0.14 ± 0.79	0.00 (0.40)	-2.10 ± 1.17	-1.90 (1.80)	0.006*
Nasal tip projection	0.10 ± 0.49	0.20 (0.90)	-0.57 ± 0.65	-0.60 (0.80	0.072
Nostril H/W ratio	-0.06 ± 0.06	-0.05 (0.08)	-0.51 ± 0.26	-0.54 (0.47)	0.001*
Columella angle (in degrees)	86.86 ± 2.67	88.00 (1.00)	82.71 ± 4.65	83.00 (5.00)	0.081^#^

Bold values indicate statistically significant differences

* Significance was set at a *p*-value < 0.05, *p* value^1^: Mann Whiteny U test, ^#^Independent t test

The intraclass correlation coefficient was applied to assess intra-examiner reliability and indicated reliable measurements (Table 5).

**Table 5:** Intra-examiner reliability of all measured parameters

Variables	ICC	95% CI	*p* value
Columella length	0.985	0.925, 0.997	< 0.001*
Nasal tip projection	0.999	0.994, 1.00	< 0.001*
Alar width at max convexity	0.986	0.933, 0.997	< 0.001*
Total alar base width	0.989	0.948, 0.998	< 0.001*

Bold values indicate statistically significant differences

*Significance was set at a *p*-value < 0.05, *ICC* Intraclass correlation coefficient

Comparison of VAS between 2 groups showed statistically significant difference (Table 6).

**Table 6:** Comparison of VAS scores between study and control groups

	Study Group	Control Group	*p *value
Mean ± SD	8.86 ± 0.66	5.07 ± 0.92	< 0.001*
Median	9.00	5.00	
Min - Max	8.00–10.00	3.00–6.00	

Bold values indicate statistically significant differences

* Significance was set at a *p*-value < 0.05, *p* value: Mann Whiteny U test

No intraoperative or postoperative harms related to the intervention were observed.

## Discussion

Achieving nasal symmetry without relapse is one of the challenging concerns for all surgeons after unilateral cleft lip nose repair. The difference in growth between normal and cleft sides is a major contributor to relapse that usually occurs in the first year postoperatively and remains stable afterwards. Managing this relapse can be managed by presurgical NAM molding, intraoperative rhinoplasty and subsequent use of postsurgical nasal conformers [11]. Some authors reported routine use presurgical NAM highlighted their value in ensuring stability of the nasal contour postoperatively [33, 34]. Nevertheless, surveys have revealed that only 37% of U.S. centers adopt NAM as part of their clinical protocol [35]. In our protocol, NAM was not utilized due to its high cost, issues with parental compliance or patients presenting beyond the optimal treatment window for NAM. Recognizing the limited accessibility and timing constraints of NAM in routine practice, postoperative nasal conformers were implemented in this pilot study as a pragmatic adjunct following primary nasal cartilage dissection to assist in maintaining nasal symmetry. Given the known impact of cleft severity on nasal support and postoperative symmetry, we retrospectively verified that baseline severity did not differ significantly between groups, reducing the likelihood that outcome differences were attributable to deformity extent rather than the intervention.

Various types of ready-made nasal conformer have been described in literature generally consisting of rubber tubes connected with a columellar bridge on bilateral basis [14]. In contrast, our design was a unilateral customized conformer to each infant with controlled uniform offset, making it comfortable and inconspicuous as described by Yuzuriha [36]. Although commercially available prefabricated nasal retainers often achieve reasonable anatomical conformity, the proposed workflow introduces potential added value through laterality-specific mirroring, controlled offset customization, and a reproducible digital fabrication process that minimizes operator-dependent variability. Moreover, in our protocol, the conformer was secured to the medial and lateral crura with two sutures and maintained for three months. This differs from Tan et al. where they reported that the conformer slipped from the infants and caused discomfort [37]. Parents were instructed to perform daily nasal cleaning using wet gauze while the sutured PMMA conformer remained in situ. With close supervision, this protocol was well tolerated, and no infections or odor-related issues were reported. Zhang Bin attempted to overcome this problem by fabricating larger sizes of nasal conformer to nostril size or fixing it by punching tape [38]. Children experienced some discomfort owing to the use of ready-made nasal conformers that failed to provide proper support as the nasal cavities are irregular in shape while exerting unfavorable tension on areas that did not require support as reported by Yuzuriha [36]. Our protocol addressed this issue where the nasal conformer followed the exact anatomical landmarks of the infants’ nasal cavities owing to its custom-made nature. Potential safety concerns related to nasal impression procedures in infants were mitigated through the use of fast-setting silicone putty, a minimal volume of material, and continuous airway supervision, with no impression-related adverse events recorded. Although PMMA used in our protocol is a rigid material, no mucosal ulceration, pressure necrosis, or clinically significant tissue irritation was observed during the retention period. This may be attributed to precise digital customization, controlled offset design to reduce pressure, careful polishing of margins, and close postoperative monitoring. In our sample, left-sided clefts were more prevalent, consistent with the findings of Begum et al. (2019) [39]. At six months, anthropometric measurements demonstrated statistically significant improvements in the study group compared to the control across all parameters. This was consistent with Al-Qatami et al. in 2022 whom they found statistically significant better results in cleft nasal dimensions regarding all measurements except nasal tip projection which was not significantly better [30]. Waewsanga et al. [40] documented no statistical difference in alar base width, nostril width and height between cleft and non-cleft side which is consistent with our results. The columella angle showed no statistically significant difference in the study group compared with the control group. This contrasts with the findings of Funayama et al., who reported significantly better results between the two groups. Furthermore, they observed a significant difference in nostril height, while nostril width showed no statistical difference. In addition, the nostril height-to-width ratio was significantly smaller in the control group than in the study group [41].

In the present study, the use of a VAS enabled quantification of parental perception of nasal symmetry after cleft lip repair. The mean VAS scores indicated significantly improved symmetry in the study group compared with the control group. These findings are in agreement with previous reports demonstrated that VAS is a sensitive measure for detecting subtle differences in nasal and facial aesthetics [42, 43].

One limitation of this study is the small sample size, highlighting the need for larger, multicenter studies to confirm these preliminary findings which should be interpreted with caution. Also future studies should compare outcomes when presurgical NAM is used routinely in combination with postsurgical nasal conformers. The limited use of NAM in this study represents an important constraint and may have influenced baseline nasal morphology and postoperative symmetry. Another limitation of this study is that it cannot serve bilateral cases. Blinding was not feasible for surgeons or parents due to the nature of the surgical intervention and the visible postoperative use of nasal conformers; however, outcome assessors were also not blinded, although this could have been implemented. This may have introduced potential assessment and response bias, also findings may be affected by variability in deformity severity. The rigidity of the PMMA conformer could be a potential limitation if the device is not carefully polished or customized to the patient’s anatomy. Additional limitations include the short follow-up period (6 months), which limits evaluation of long-term nasal growth. We recommend long-term follow-up studies to assess the effects of conformer use on facial appearance and patient quality of life. Future studies should include direct comparisons with prefabricated nasal retainers to better define the relative clinical benefits of customized conformers.

## Conclusion

Customized nasal conformers, when employed in unilateral cleft lip repair, showed promising potential in reducing relapse, improving nasal symmetry, refining nasolabial esthetics, and enhancing parental satisfaction.

## Data Availability

The data used and analyzed in this study are available from the corresponding author upon reasonable request.

## Abbreviations

CLP: Cleft lip and palate
NAM: Nasoalveolar molding
SPSS: Statistical Package for Social Science
VAS: Visual Analogue Scale
PMMA: Polymethyl methacrylate
STL: Standard Tessellation Language
